# Extraesophageal reflux and reflux aspiration in dogs with respiratory diseases and in healthy dogs

**DOI:** 10.1111/jvim.16622

**Published:** 2023-01-19

**Authors:** Sirkku Kouki, Sanna J. Viitanen, Ninna Koho, Henna P. Laurila, Liisa Lilja‐Maula, Saila Holopainen, Mikko Neuvonen, Mikko Niemi, Aline Fastrès, Cécile Clercx, Minna M. Rajamäki

**Affiliations:** ^1^ Department of Equine and Small Animal Medicine, Faculty of Veterinary Medicine University of Helsinki Helsinki Finland; ^2^ Department of Clinical Pharmacology, Faculty of Medicine University of Helsinki and Helsinki University Hospital Helsinki Finland; ^3^ Department of Clinical Sciences, Faculty of Veterinary Medicine University of Liège Liège Belgium

**Keywords:** aerodigestive disorder, bile acid, canine, canine idiopathic pulmonary fibrosis, mass spectrometry

## Abstract

**Background:**

Salivary bile acids are used to diagnose extraesophageal reflux (EER) and to evaluate the risk of reflux aspiration that is associated with respiratory diseases in dogs.

**Objectives:**

To study total bile acid (TBA) concentrations in saliva and in bronchoalveolar lavage fluid (BALF) to investigate EER and reflux aspiration in dogs with respiratory diseases and in healthy dogs.

**Animals:**

Thirty‐one West Highland White Terriers (WHWTs) with idiopathic pulmonary fibrosis (IPF), 12 dogs with inflammatory airway disease (IAD), 6 dogs with recurrent pneumonia (RP), 26 brachycephalic dogs (BD), 27 healthy WHWTs (HW), 52 healthy dogs (HD). All privately‐owned dogs.

**Methods:**

Saliva and BALF were collected from dogs in each group.

**Results:**

Salivary TBA concentrations were higher in IPF (median 0.1692 μM, interquartile range [IQR] 0.1115‐0.2925 μM, Cohen's *d* 3.4, 95% confidence interval [CI] 2.2‐4.0, *P* < .001) and BD (0.0256 μM, IQR 0.0086‐0.0417 μM, *d* 0.5, CI −0.1 to 1.1, *P* = .003) compared to HD (0 μM, IQR not quantifiable [n.q.]‐0.0131 μM). Bronchoalveolar lavage fluid TBA concentrations were higher in IPF (0.0117 μM, IQR 0.0048‐0.0361 μM, *d* 0.5, CI 0‐1.1, *P* < .001) compared to HD (0 μM, IQR n.q.‐0.0074 μM).

**Conclusion and Clinical Importance:**

Extraesophageal reflux and reflux aspiration occur in healthy dogs and those with respiratory diseases.

**Abbreviations**
BALF: bronchoalveolar lavage fluid
BD: brachycephalic dogs
BOAS: brachycephalic obstructive airway syndrome
CI: confidence interval
EER: extraesophageal reflux
GER: gastroesophageal reflux
HD: healthy other breed dogs
HDpost: healthy other breed dogs' postfeeding saliva samples
HDpre: healthy other breed dogs' prefeeding saliva samples
HRCT: high‐resolution computed tomography
HW: healthy West Highland White Terriers
IAD: inflammatory airway disease
IPF: idiopathic pulmonary fibrosis
IQR: interquartile range
MA: microaspiration
RP: recurrent pneumonia
TBA: total bile acid
VFSS: videofluoroscopic swallow study
WHWT: West Highland White Terrier

## 1 INTRODUCTION

Gastroesophageal (GER) and extraesophageal reflux (EER) are defined as the return of gastroduodenal contents coming back to the esophagus and in EER further to the laryngopharyngeal area.^1^, ^2^ Reflux occurs in dogs with gastrointestinal disorders such as in megaesophagus.^3^ In people, GER and EER are linked to the pathogenesis and progression of several acute and chronic respiratory diseases and are frequently reported with gastrointestinal conditions.^4^, ^5^, ^6^, ^7^, ^8^, ^9^, ^10^, ^11^, ^12^, ^13^, ^14^ These aerodigestive disorders, referring to a connection between respiratory and upper digestive tract conditions, include a wide range of common diseases, such as asthma, chronic pulmonary obstructive disease, and idiopathic pulmonary fibrosis (IPF).^11^, ^15^, ^16^ The association between reflux and respiratory diseases is only partly evaluated in dogs but suggests a similar connection.^17^, ^18^, ^19^, ^20^, ^21^, ^22^, ^23^

Gastroesophageal reflux is suspected to aggravate lower respiratory diseases: by refluxate microaspiration (MA) and by stimulating the sensitized esophageal‐bronchial neuronal pathway.^24^, ^25^ In MA, droplets of gastroduodenal contents are inhaled to the respiratory tract where they cause irritation and further inflammation to the airways and parenchyma.^1^ Reflux occurs in asymptomatic dogs based on nuclear scintigraphy and videofluoroscopic swallow studies (VFSS).^18^, ^26^, ^27^, ^28^ Pathologic and physiologic reflux are suspected to have differences in volume, timing, and location of the refluxate within the esophagus.^18^, ^26^, ^27^, ^28^ Ability to detect abnormal reflux and MA in dogs with respiratory diseases can shed light on novel treatment modalities. In certain human respiratory diseases, GER medications—H2‐blockers or proton‐pump inhibitors—are suggested to reduce symptoms and prolong survival time, but there is no consensus regarding the benefit of acid suppressants in the treatment of respiratory conditions.^25^, ^29^, ^30^, ^31^, ^32^

Gastroduodenal contents, such as bile acids and pepsin, in bronchoalveolar lavage fluid (BALF) can be used to diagnose reflux aspiration.^12^, ^33^, ^34^, ^35^, ^36^ Measuring these from saliva samples has the potential to work as an easy, noninvasive biomarker for assessing EER and the risk of MA.^12^, ^33^, ^34^, ^35^, ^36^ In dogs, previously we found bile acids in BALF in respiratory diseases and in healthy West Highland White Terriers (WHWT) but not in healthy Beagles.^20^ The WHWT breed is predisposed to a chronic, progressive lung disease, IPF in dogs, with unknown etiology and pathogenesis.^37^, ^38^, ^39^, ^40^ Idiopathic pulmonary fibrosis in dogs shares clinical and pathological similarities with IPF in humans in which MA is suggested to be a possible etiological or aggravating factor.^12^, ^41^

The aim of this cross‐sectional observational study was to evaluate the presence and concentration of bile acids in saliva and in BALF in dogs with respiratory diseases and in healthy dogs. We hypothesized that EER occurs in dogs with various respiratory diseases, that MA can be detected in diseased dogs, and that EER is associated with MA. We also hypothesized that feeding increases salivary bile acids.

## 2 MATERIALS AND METHODS

### 2.1 Study subjects

A total of 154 privately‐owned pet dogs were included in this cross‐sectional observational study under the umbrella of the WHWT IPF project performed at the Veterinary Teaching Hospitals of University of Helsinki, Finland, and University of Liège, Belgium. Saliva samples were prospectively collected from 74 dogs: 6 WHWTs with IPF, 9 dogs with inflammatory airway disease (IAD), 4 dogs with recurrent pneumonia (RP), 20 healthy brachycephalic dogs (BD), 10 healthy WHWTs (HW), and 25 healthy other breed dogs (HD). From these, paired BALF samples collected on the same day were available from 9 IAD, 4 RP, and 4 HD. The rest of BALF samples consisted of both banked and prospectively collected samples from 80 dogs: 25 WHWTs with IPF, 3 IAD, 2 RP, 6 BD, 17 HW, and 27 HD. Most of the dogs with BALF samples have participated in other studies,^42^, ^43^, ^44^, ^45^, ^46^ excluding the study of Määttä et al. measuring BALF bile acid concentrations.^20^ Pregnant or lactating dogs and dogs under the age of 6 months were excluded. Use of H2‐blockers or proton‐pump inhibitors was considered as an exclusion criterion.

Disease history was obtained from owners and none of the dogs had had signs of acute or chronic gastrointestinal diseases (ie, vomiting, diarrhea, anorexia) in the 6 months before sample collection. Physical examination was performed, and serum biochemistry and hematology were analyzed in all dogs except in HD with only saliva sample. In serum biochemistry and hematology, only incidental changes were detected. Fecal analyses for parasites were examined using Baermann and flotation methods for IAD dogs, RP dogs, 7/31 of IPF WHWTs, and 10/27 of HW and no parasites were found. Thoracic high‐resolution computed tomography (HRCT) or radiographs were available from all diseased dogs. In HW group, HRCT was available from 22/27, thoracic radiographs from 4/27, and postmortem histopathology examination of lung tissue from 1/27 of the dogs. Arterial blood gas analyses were analyzed from all of IAD dogs, all of RP dogs, 30/31 of IPF WHWTs, and 16/27 of HW.

Inclusion criteria were defined separately for each group. The diagnosis of IPF was based on all or most of the following: compatible history with signs such as exercise intolerance and cough, typical findings in clinical examination including Velcro crackles in lung auscultation, hypoxemia in arterial blood gas analyses, and characteristic findings in thoracic HRCT or postmortem IPF findings in histopathology.^37^, ^42^, ^43^, ^44^, ^45^ West Highland White Terriers were considered healthy when they had no signs of respiratory disease, such as cough, and no abnormal findings in physical examination or in thoracic HRCT or radiographs or postmortem examination and no hypoxemia.

Dogs with IAD had a history of cough (duration >2 months during a year), airway inflammation for which parasitic, infectious, neoplastic and cardiac causes were excluded, over 6% of neutrophils, lymphocytes or eosinophils in BALF, or a mixed inflammatory cytologic pattern that lacked intracellular bacteria, and no growth of potential pathogens on microbial culture.

A group of dogs with RP and possible aspiration etiology were included in the study. The group comprised of 6 dogs, which were referred for further examinations because of RP. These dogs had reportedly experienced median of 5 pneumonias documented at the referring veterinarian. All of these dogs were examined in between pneumonia episodes, when the dogs were clinically stable. Three of them were being administered enrofloxacin at the time of examination. In all RP dogs, thorough clinical examinations including laryngeal evaluation, bronchoscopy, and BALF sampling were performed. Additionally, VFSS, ciliary electron microscopy, and postmortem examination were performed in selected cases. None of the dogs had alveolar density or bacterial growth in BALF samples at the time of examination. The following possible predisposing conditions for RP were identified: esophageal dysmotility (n = 1), bronchiectasis (n = 1).

The brachycephalic group was composed of English Bulldogs participating in an English Bulldog health study (20/26, saliva samples), conducted at the University of Helsinki, Finland, and English and French Bulldogs (6/26, BALF samples) examined at the University of Liège, Belgium.^46^, ^47^, ^48^ All dogs were considered healthy by their owners and had no findings of respiratory disease barring signs of brachycephalic obstructive airway disease.

All HD were considered healthy by their owners. The dogs with saliva samples had had no signs of respiratory or gastrointestinal disease (ie, cough, exercise intolerance, diarrhea, vomiting) for the 6 months before sample collection. Healthy other breed dogs with BALF samples were considered healthy based on physical examination, hematology and serum biochemistry, bronchoscopy, and BALF cytology.^48^

### 2.2 Sample collection

Saliva samples were collected using a commercially available method (Salivette collection tube, Sartstedt, Germany) after 12 hours of fasting. In HD, postfeeding samples (HDpost) were obtained 1 hour after feeding from 19/25 HD (HDpre). An absorbent cotton swab was placed into the dog's mouth for at least approximately 30 seconds or for longer depending on the wetting of the swab. The saliva was separated from the swab by centrifugation (20 minutes, 2200 g) and stored at −112°F (−80°C).

Bronchoalveolar lavage fluid samples were collected during bronchoscopy from left and right caudal lung lobes with 1‐2 mL/kg of sterile saline per lobe once or twice.^49^, ^50^ In RP dogs, the affected lobe was selected based on radiographs, HRCT or accessibility and the amount of saline used was 1 mL/kg or a total of 40 mL twice per lobe.^51^, ^52^ The supernatant was separated by centrifugation (10 minutes, 100 g) and stored at −112°F (−80°C).

### 2.3 Bile acid concentration analysis

Bile acid concentration analyses were run in the laboratory HUSLAB, Helsinki University Central Hospital, by high performance liquid chromatography‐tandem mass spectrometry.^53^ A total of 17 different bile acids were measured and then summed as a total bile acid (TBA) concentration. Measurements were carried out by a Nexera X2 UPLC system (Shimazdu, Kyoto, Japan) coupled to a 5500 Qtrap mass spectrometer interfaced with an electrospray ion source (ABSciex, Toronto, Ontario, Canada) as described before.^20^

Because of technical details in analyses the limit of quantification was 0.005 μM for ursodeoxycholic acid, glycochenodeoxycholic acid, hyodeoxycholic acid, glycocholic acid, glycolithocholic acid, chenodeoxycholic acid, glycoursodeoxycholic acid, deoxycholic acid, glycodeoxycholic acid, lithocholic acid, taurolithocholic acid, taurochenodeoxycholic acid, taurocholic acid, tauroursodeoxycholic acid, and taurohyodeoxycholic acid. For taurodeoxycholic acid, the limit of quantification was 0.0025 μM, and for cholic acid, 0.01 μM.

### 2.4 Statistical analysis

All statistical analyses were performed by SAS System for Windows, version 9.4 (SAS Institute, Inc, Cary, North Carolina). Data was transformed logarithmically to satisfy the normality‐assumption. When the TBA concentration was below the limit of quantification, the observed zero‐concentrations were imputed as half of the limit of quantification. The differences between TBA in IPF, BD, IAD, RP, and healthy WHWTs against TBA in HD and the differences between TBA in BD, IAD, RP, and healthy WHWTs against IPF were analyzed with analysis of variance in both saliva and BALF samples. The models included the dog group as the sole fixed effect. Dunnett's test was used to adjust for multiplicity. For the HD group, the pre‐ and postfeeding (HDpre and HDpost) measurements were compared with a paired *t* test. The Cohen's *d* (*d*) effect size and its associated 95% confidence interval (95% CI) was calculated. *P*‐values <.05 were considered statistically significant in all analyses. For the dogs where both BALF and saliva measurements were conducted, the measurements within dog were analyzed descriptively with frequency tables (bile acids detected [yes/no]).

## 3 RESULTS

### 3.1 Study subjects

A total of 93 saliva samples from 74 dogs including prefeeding (n = 74) and postfeeding (n = 19) samples and BALF samples from 97 dogs were analyzed. The demographics including sex, age, and breed of the dogs are listed in Supporting Information (Tables S1 and S2).

### 3.2 Saliva samples

**FIGURE 1:**
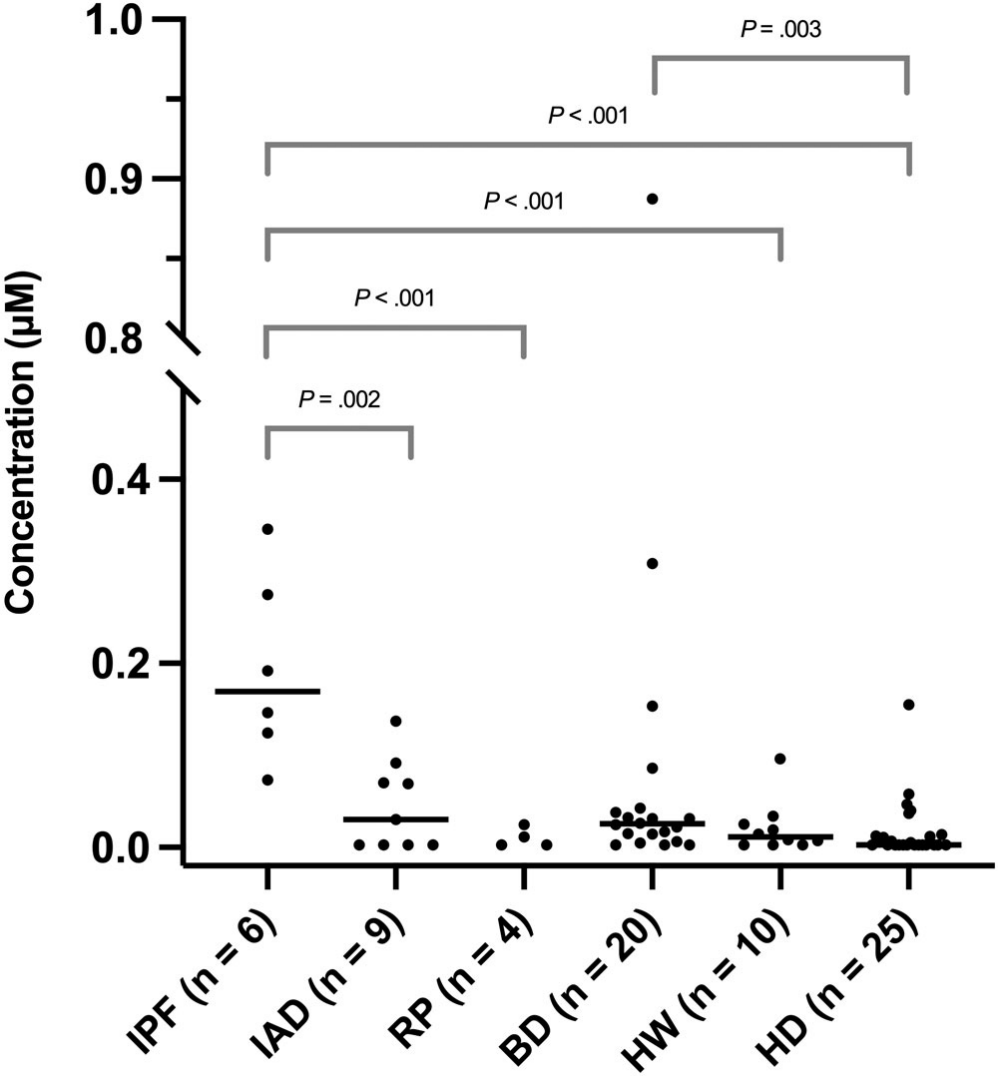
Total bile acid (TBA) concentrations in saliva samples of different dog groups. Statistically significant differences (*P* < .05) between TBA in other groups against TBA in dogs with idiopathic pulmonary fibrosis (IPF) and healthy other breed dogs (HD) are marked. The horizontal lines indicate median TBA values. BD, brachycephalic dogs; IPF, idiopathic pulmonary fibrosis; HD, healthy other breed dogs; HW, healthy West Highland White Terriers; IAD, dogs with inflammatory airway disease; RP, dogs with recurrent pneumonia

In saliva samples, concentrations of TBA were above the limit of quantification in 100% of WHWTs with IPF (6/6, 95% binomial 95% CI 54%‐100%), 56% of IAD (5/9, CI 21%‐86%), 50% of RP (2/4, CI 7%‐93%), 85% of BD (17/20, CI 62%‐97%), 70% of HW (7/10, CI 35%‐93%), and 48% of HD (12/25, CI 34%‐63%). Salivary TBA concentrations in different groups are presented in Figure 1. Compared to HD (median 0 μM, interquartile range [IQR] not quantifiable [n.q.]‐0.0131 μM), concentrations of TBA were significantly higher in IPF WHWTs (0.1692 μM, IQR 0.1115‐0.2925 μM, *d* 3.4, 95% CI 2.2‐4.0, *P* < .001) and BD (0.0256 μM, IQR 0.0086‐0.0417 μM, *d* 0.5, 95% CI −0.1 to 1.1, *P* = .003). In WHWTs with IPF, TBA concentrations were significantly higher compared to all other groups: IAD (0.0305 μM, IQR n.q.‐0.0808 μM, *d* 2.0, 95% CI 0.7‐3.3, *P* = .002), RP (0.0058 μM, IQR n.q.‐0.0215 μM, *d* 2.3, 95% CI 0.7‐3.9, *P* < .001), BD (*d* 0.6, 95% CI −0.4 to 1.5, *P* = .004), and HW (0.0115 μM, IQR n.q.‐0.0275 μM, *d* 2.6, 95% CI 1.3‐4.0, *P* < .001). No statistically significant differences were found between IAD and HD (*d* − 0.74, 95% CI −1.5 to 0, *P* = .11), RP and HD (*d* 0.2, 95% CI −0.8 to 1.3, *P* = .97), and HW and HD (*d* 0.1, 95% CI −0.9 to 0.6, *P* = .36).

**FIGURE 2:**
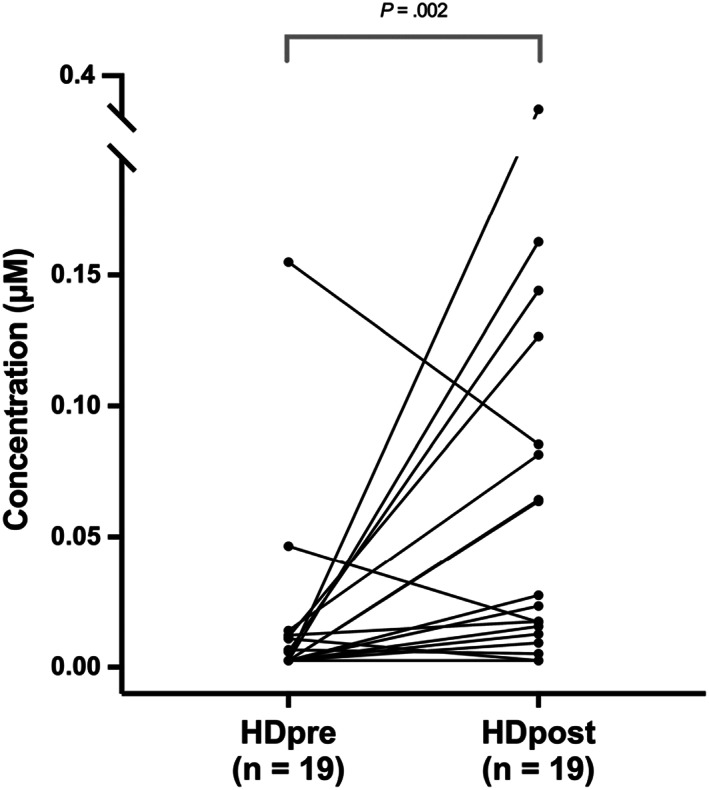
Total bile acid (TBA) concentrations in paired saliva samples of healthy other breed dogs before feeding (HDpre) and after feeding (HDpost)

In HD, prefeeding salivary TBA concentrations (0 μM, IQR n.q.‐0.155 μM) were significantly lower compared to postfeeding samples (0.0235 μM, IQR n.q.‐0.3601 μM, *d* 0.75, 95% CI 0.9‐1.4, *P* = .002; Figure 2).

### 3.3 Bronchoalveolar lavage fluid samples

**FIGURE 3:**
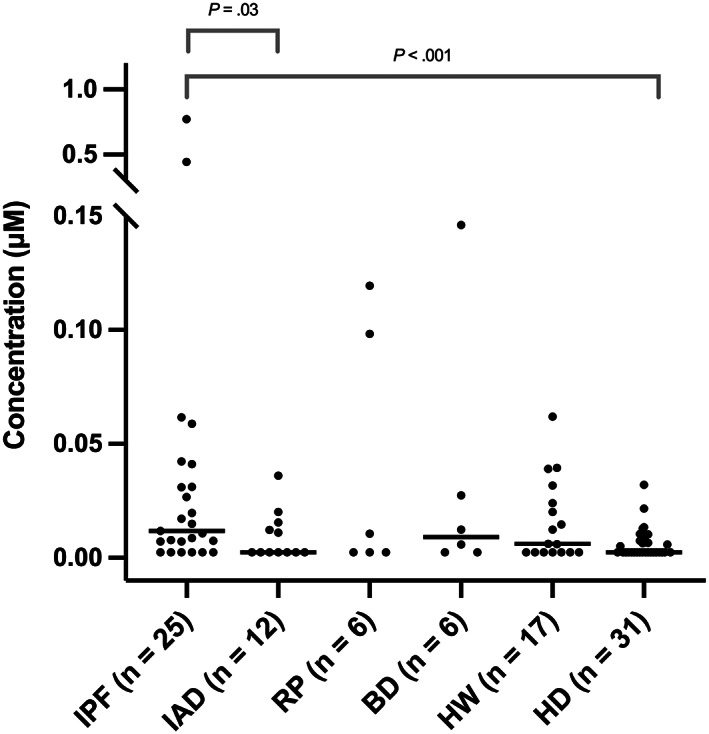
Total bile acid (TBA) concentrations in bronchoalveolar lavage fluid (BALF) samples of different dog groups. Statistically significant differences (*P* < .05) between TBA in other groups against TBA in dogs with idiopathic pulmonary fibrosis (IPF) and healthy other breed dogs (HD) are marked. The horizontal lines indicate median TBA values. BD, brachycephalic dogs; IPF, idiopathic pulmonary fibrosis; HD, healthy other breed dogs; HW, healthy West Highland White Terriers; IAD, dogs with inflammatory airway disease; RP, dogs with recurrent pneumonia

In BALF samples, concentrations of TBA were above the limit of quantification in 76% of WHWTs with IPF (19/25, CI 55%‐91%), 42% of IAD (5/12, CI 15%‐72%), 50% of RP (3/6, CI 12%‐88%), 59% of HW (10/17, CI 33%‐82%), 67% of BD (4/6, CI 22%‐96%) and 42% of HD (13/31, CI 25%‐61%). Bronchoalveolar lavage fluid TBA concentrations in different groups are presented in Figure 3. Compared to HD (median 0 μM, IQR n.q.‐0.0074 μM), TBA concentrations were significantly higher in dogs with IPF (0.0117 μM, IQR 0.0048‐0.0361 μM, *d* 0.5, 95% CI 0‐1.1, *P* < .001). In IPF, TBA concentrations were also significantly higher than in IAD (0 μM, IQR n.q.‐0.0147 μM, *d* 0.4, 95% CI −0.4 to 1.2, *P* = .03). No statistically significant differences were found between HD and IAD (*d* − 0,4, CI −1.0 to 0.3, *P* = .58), HD and RP (0.005 μM, IQR 0‐0.1306 μM, *d* − 1.5, 95% CI −2.5 to −0.6, *P* = .10), HD and BD (0.009 μM, IQR n.q.‐0.0570 μM, *d* − 1.2, 95% CI −2.1 to −0.3, *P* = .10), HD and HW (0.006 μM, IQR n.q.‐0.0570 μM, *d* − 1.2, 95% CI −2.1 to −0.3, *P* = .06), BD and IPF (*d* − 0.2, 95% CI −1.1 to 0.7, *P* = .59), and HW and IPF (*d* 0.4, 95% CI −1 to 0.2, *P* = .22).

### 3.4 Paired saliva and bronchoalveolar lavage fluid samples

**FIGURE 4:**
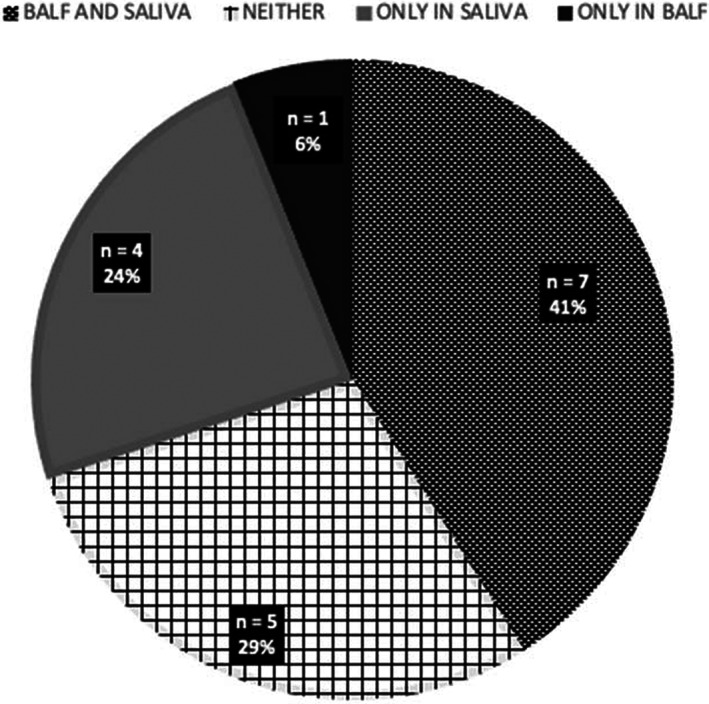
Presence of bile acids in paired saliva and bronchoalveolar lavage fluid samples (n = 17)

In dogs where both saliva and BALF samples were obtained at the same appointment (n = 17), the presence of bile acids in saliva and BALF samples are presented in Figure 4. In 71% of the dogs (12/17, 95% binomial CI 44%‐90%), the presence or absence of bile acids in saliva did predict the presence or absence of bile acids in BALF.

## 4 DISCUSSION

This study was designed to elucidate the presence of EER and MA in healthy dogs and in those with various respiratory diseases by measuring bile acids from saliva and BALF samples. The results indicate that EER occurs in both healthy dogs and those with a variety of respiratory diseases, as bile acids were present in saliva of dogs from all groups. When comparing concentrations in different groups, however, bile acid concentration was highest concentration in IPF dogs followed by BD considered healthy by their owner. Timing of saliva collection is important as we found that feeding increases salivary TBA concentrations compared to fasted dog. Using new, different cohorts—both WHWTs and other dogs—compared to our previous BALF bile acid study, it was found again that TBA concentrations in IPF WHWTs' BALF samples were significantly higher than in healthy dogs.^20^ Results of this study also suggest that the presence or absence of salivary bile acids is associated with simultaneous bile acid detection in BALF in the majority of dogs.

By measuring salivary bile acids, we studied EER and risk for MA. Aerodigestive disorders describing the complex relationship between airway protection failures and abnormal swallowing are poorly recognized in dogs because of inadequate clinical recognition, limitations in available diagnostics, and lack of thorough studies.^11^, ^15^, ^16^, ^17^, ^19^, ^26^ By detecting bile acids in saliva, it was noticed that EER occurs both in healthy dogs and dogs with respiratory diseases. Previous studies using VFSS, scintigraphy, and ambulatory esophageal pH monitoring have described GER,^17^, ^18^, ^27^, ^28^, ^54^, ^55^ and scintigraphy and proteomic characterization studies EER,^27^, ^56^ in healthy and diseased dogs. This study supports the insight that EER and GER can also be physiological without apparent clinical consequence, as is noted in humans.^5^, ^6^, ^7^, ^26^ However, in dogs, mild and intermittent clinical signs, such as heartburn and nausea in people, can be unrecognizable and it is possible that some dogs in the HD group have reflux related clinical signs without owners noticing them.

Salivary bile acids were found in all groups of dogs with respiratory disease. Nevertheless, IPF WHWTs differed from other groups as their bile acid concentrations were significantly higher compared to all the other groups. Similarly, people with IPF seem to have more salivary bile acids compared to healthy controls and to patients with other interstitial lung diseases.^12^ Extraesophageal reflux was detected in all IPF WHWTs whereas only half of HD had even a detectable amount of it, which suggests that pathological reflux might differ in frequency and volume compared to physiological reflux. Establishing the differences between pathological and physiological reflux in dogs and their effect on MA can help clarifying the etiological and aggravating factors of IPF. Early clinical signs of IPF develop slowly, which results in dogs being diagnosed when the disease is already advanced.^39^ Detection of EER as a risk factor for MA and thereby potentially for IPF evolution could help in finding WHWTs at risk for IPF earlier. It can also lead to novel treatment modalities, as has happened in IPF in humans where antireflux therapy—H2‐blockers or proton‐pump inhibitors—is used to delay disease progression.^57^

In the BD group, salivary TBA concentrations were significantly higher than in healthy other breed dogs. Brachycephalic dogs were all considered healthy by their owners. However, in clinical examination, they were found to have signs of brachycephalic obstructive airway syndrome (BOAS) of varying severity, as previously described by Lilja‐Maula et al.^46^ Effect of BOAS severity on salivary TBA concentration was not examined in these dogs. The high TBA concentrations are not surprising, because the prevalence of different esophageal and gastrointestinal abnormalities, such as hiatal hernias, is reported to be high in brachycephalic dogs.^23^, ^54^, ^58^, ^59^, ^60^ These common abnormal conditions include GER, which is believed to be partially caused by high intrathoracic pressure generated to overcome upper respiratory tract obstruction.^61^, ^62^ Distal esophagitis is also a common finding in BD, suggesting GER to be chronic and frequent.^23^, ^63^ The severity of clinical gastrointestinal signs, including dysphagia, regurgitation, vomiting and ptyalism, is also likely to be related to the severity of BOAS.^23^, ^64^ BD with clinical and histopathological evidence of gastrointestinal conditions do not always show associated clinical signs.^23^, ^65^ It is noteworthy that even if they did, owners might not always recognize these clinical signs or consider them abnormal.

Interrelationship between EER and MA was studied by investigating if the presence of salivary bile acids was associated with simultaneous bile acid detection in BALF. In 71% (12/17) of dogs the presence or absence of salivary bile acids was similar to the detection of bile acids in BALF samples. This indicates that the presence of bile acids in saliva could serve as a potential predictor of MA. Saliva collection has several advantages as it is an easy, quick, and noninvasive procedure compared to BALF sampling which requires general anesthesia, specific equipment, and expertise. Other methods for reflux aspiration risk evaluation include VFSS and scintigraphy. VFSS is the most commonly used method for GER and EER evaluation in dogs and can be done without anesthesia but lacks sensitivity to detect reflux with MA.^28^, ^54^, ^66^, ^67^, ^68^, ^69^ Nuclear scintigraphy, then again, is successfully used to document reflux, also intermittent and nonacidic, and aspiration in people^70^, ^71^, ^72^ and in dogs, but is thus far limited to research settings in dogs.^27^

Feeding's effect on saliva's TBA concentration was also evaluated to establish the optimal timing for sampling relative to feeding. Fed dogs had significantly higher TBA levels compared to 12‐hour‐fasted dogs, possibly because of increased EER or excretion of bile or both, which indicates that sampling time is a decisive factor when measuring salivary TBA concentrations. The impact of diet in humans, including its composition, timing, and the size of a meal is a well‐known contributor to both physiological and pathological reflux.^73^, ^74^, ^75^, ^76^ In this study, it was not explored whether the composition of the dogs' diet or size of the meal influenced TBA concentration.

Microaspiration occurs in various respiratory diseases of dogs and also in HW.^20^ In this study, using new cohorts of WHWTs in both groups, TBA concentrations in IPF WHWTs' BALF samples were significantly higher than in healthy other breed dogs. No statistical difference between IPF WHWTs and HW was found. Similarly, as shown in a larger cohort, these findings suggest that EER and MA might be contributing aggravating factors in the IPF pathogenesis and possibly even etiological factors of IPF.^20^ This finding of reflux aspiration concurs well with detection of high abundance of food and water contaminating bacteria in IPF and healthy WHWTs' lung microbiota compared with other healthy domestic dogs.^48^

The results do not reveal a statistically significant difference in BALF TBA concentration between RP group and HD group. However, aspiration‐related pathogenesis in lung infections is noted in microbiological studies where they are commonly caused by opportunistic bacteria belonging to normal oropharyngeal flora.^77^, ^78^ None of the dogs in this study had aspiration pneumonia, which is the most commonly recognized aerodigestive disorder in dogs, typically associated with aspiration of larger amount of gastroduodenal or foreign material.^79^, ^80^ Repetitive MA, nevertheless, could be causing continuous irritation to the airways and parenchyma and therefore could potentially be a predisposing factor for secondary bacterial infection. Three of the RP dogs were being treated with enrofloxacin at the time of the examination and because enrofloxacin is associated with high frequency of antibiotic‐associated signs of gastrointestinal dysfunction, such as vomiting and diarrhea,^81^ it cannot be ruled out if that salivary or BALF TBA concentration was increased during antibiotic treatment in these dogs.

In 42% of healthy dogs (13/31), the TBA concentration was above the limit of quantification in BALF, suggesting that MA events occur in dogs without clinical signs indicating respiratory disease. We hypothesized that healthy dogs would not aspirate because aspiration has not been identified in healthy dogs in previous bile acid, VFSS, and scintigraphy studies.^20^, ^27^, ^28^ In microbiological studies, however, bacteria belonging to normal oropharyngeal flora have been isolated from tracheal wash and lung biopsies in healthy dogs.^82^, ^83^, ^84^ In this study, bacterial culture was not performed on BALF samples in HD group. In humans, silent aspiration is a well described phenomenon in healthy adults with no clinical consequences, and only repetitive and chronic MA is considered to indicate underlying pathology.^85^, ^86^, ^87^ Further studies evaluating MA in healthy dogs is warranted to objectively classify physiological and pathological MA and their prevalence in larger population.

The small number of dogs in some groups limits this study. Despite this limitation, our study indicates that EER and MA occur in both healthy dogs and dogs with respiratory disease. It also suggests that measuring TBA in saliva has the potential as a biomarker for assessing EER and the risk of MA and that WHWTs with IPF and brachycephalic dogs are at higher risk compared to healthy dogs.

## CONFLICT OF INTEREST DECLARATION

Authors declare no conflicts of interest.

## OFF‐LABEL ANTIMICROBIAL DECLARATION

Authors declare no off‐label use of antimicrobials.

## INSTITUTIONAL ANIMAL CARE AND USE COMMITTEE (IACUC) OR OTHER APPROVAL DECLARATION

The study protocol was approved by the Committee of Experimental Animals of Southern Finland (no: ESAVI/10906/04.10.07/2017), by the Ethical Committee of the University of Liège (no: 1435 and 2245), and by the University of Helsinki Viikki Campus Research Ethics Committee (5B/2008, 13/2020, 15/2020).

## HUMAN ETHICS APPROVAL DECLARATION

Authors declare human ethics approval was not needed for this study.

## Supporting information


**TABLE S1.** Demographics of dogs included in the study.

**TABLE S2.** The dog breeds in dog groups consisting several breeds.
