# Supplementary material for: Extraesophageal reflux and reflux aspiration in dogs with respiratory diseases and in healthy dogs
Source: J Vet Intern Med. 2023 Jan 19;37(1):268–76. doi: 10.1111/jvim.16622 (PMC9889628; doi:10.1111/jvim.16622)
Supplement: Supplementary file 1 — TABLE S1. Demographics of dogs included in the study. [file JVIM-37-268-s001.pdf]

Supporting Information 1.

Table S1. Demographics of dogs included in the study.

	IPF (n = 31)	IAD (n = 12)	RP (n = 6)	BD (n = 26)	HW (n = 27)	HD (n = 52)
**Sex**						
Female	20	5	3	12	10	31
Male	11	7	3	14	17	21
**Age (years)**						
Range (median, interquartile range IQR)	7.4–14.4 (12.3, 10.4–12.8)	1.6–13.1 (8.6, 2.0–10.2)	1.0–11.7 (6.5, 1.1–10.2)	1.4–8.7 (3.0, 2.5–4.4)	0.8–14.9 (10.0, 6.5–11.4)	0.5–13.6 (3.8, 2.3–8.1)

Abbreviations: IPF, idiopathic pulmonary fibrosis; IAD, dogs with inflammatory airway disease; RP, dogs with recurrent pneumonia; BD, brachycephalic dogs; HW, healthy West Highland White Terriers; HD, healthy other breed dogs.
