# Supplementary material for: Extraesophageal reflux and reflux aspiration in dogs with respiratory diseases and in healthy dogs
Source: J Vet Intern Med. 2023 Jan 19;37(1):268–76. doi: 10.1111/jvim.16622 (PMC9889628; doi:10.1111/jvim.16622)
Supplement: Supplementary file 2 — TABLE S2. The dog breeds in dog groups consisting several breeds. [file JVIM-37-268-s002.pdf]

Supporting Information 2.

Table S2. The dog breeds in dog groups consisting several breeds.

Group	Breeds
Inflammatory airway disease (n = 12)	1 Cavalier King Charles Spaniel<br>1 Dachshund<br>1 Finnish Lapphound<br>1 Ibizan Hound<br>1 Kooikerhondje<br>1 Miniature Pinscher<br>1 Mixed breed<br>1 Rottweiler<br>1 Schipperke<br>2 Shetland Sheepdog<br>1 Toy Poodle
Recurrent pneumonia (n = 6)	1 German Shepherd<br>1 Golden Retriever<br>2 Irish Wolfhound<br>1 Miniature Schnauzer<br>1 West Highland White Terrier
Brachycephalic dogs (n = 26)	21 English Bulldog<br>5 French Bulldog
Healthy other breed dogs (n = 52)	1 American Staffordshire Terrier<br>10 Australian Shepherd<br>1 Basenji

	2 Beauceron<br><br>4 Belgian Shepherd Malinois<br><br>5 Border Collie<br><br>2 Cavalier King Charles Spaniel<br><br>1 Cocker Spaniel<br><br>1 Dachshund<br><br>1 Dalmatian<br><br>11 Labrador Retriever<br><br>1 Lapponian Herder<br><br>2 Mixed Breed<br><br>1 Mudi<br><br>1 Newfoundland Dog<br><br>1 Pumi<br><br>1 Rottweiler<br><br>1 Siberian Husky<br><br>2 Spanish Waterdog<br><br>1 Welsh Springer Spaniel<br><br>1 White Swiss Shepherd Dog
